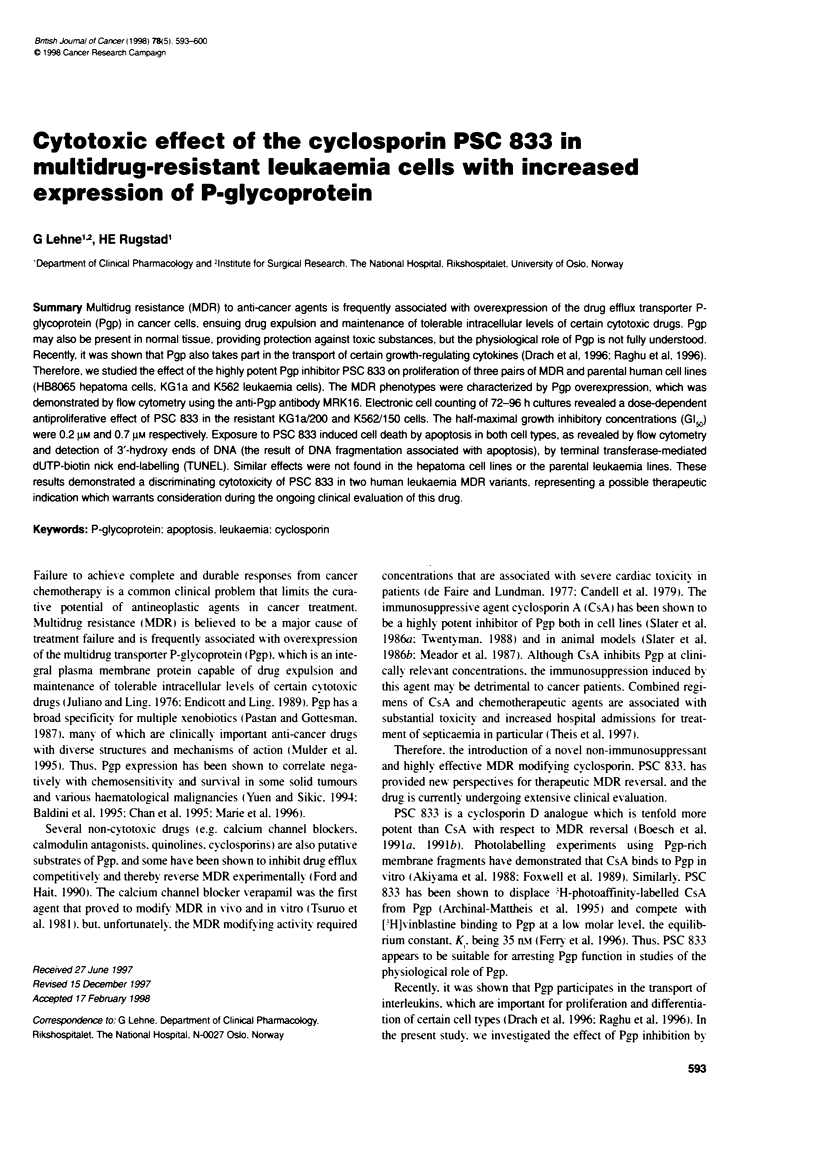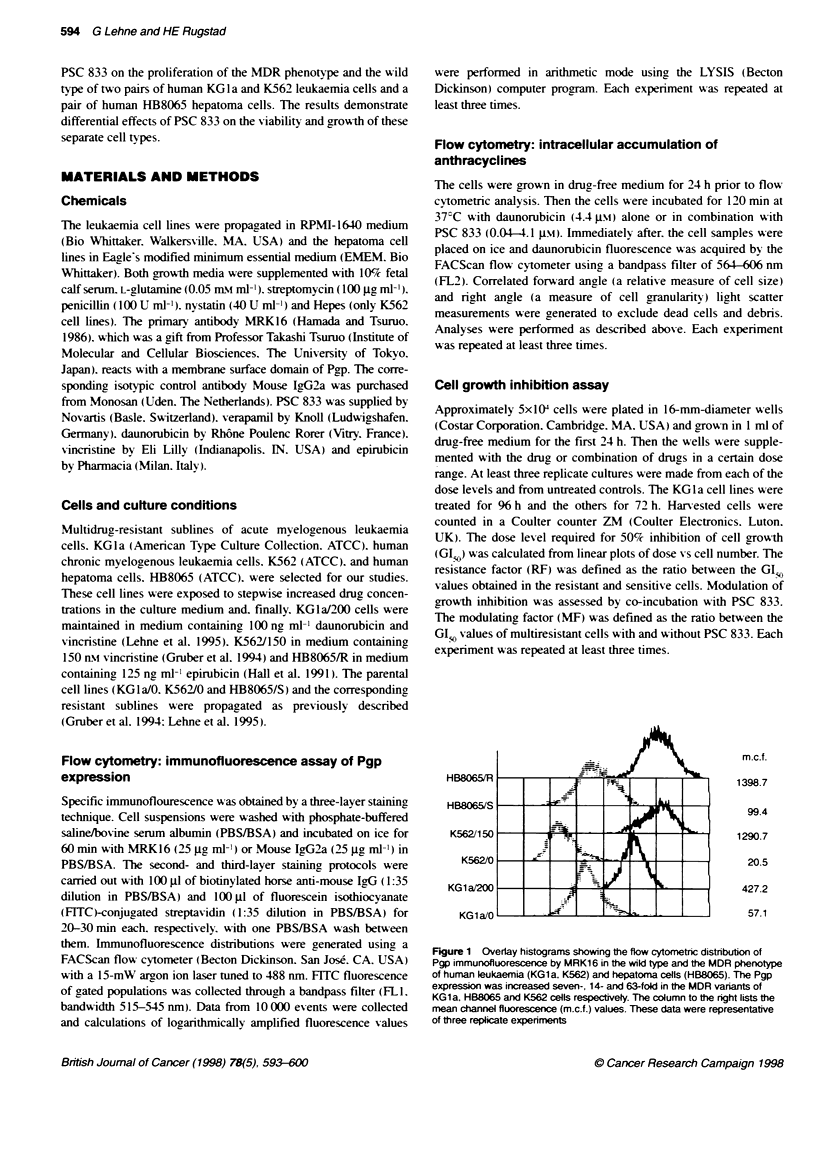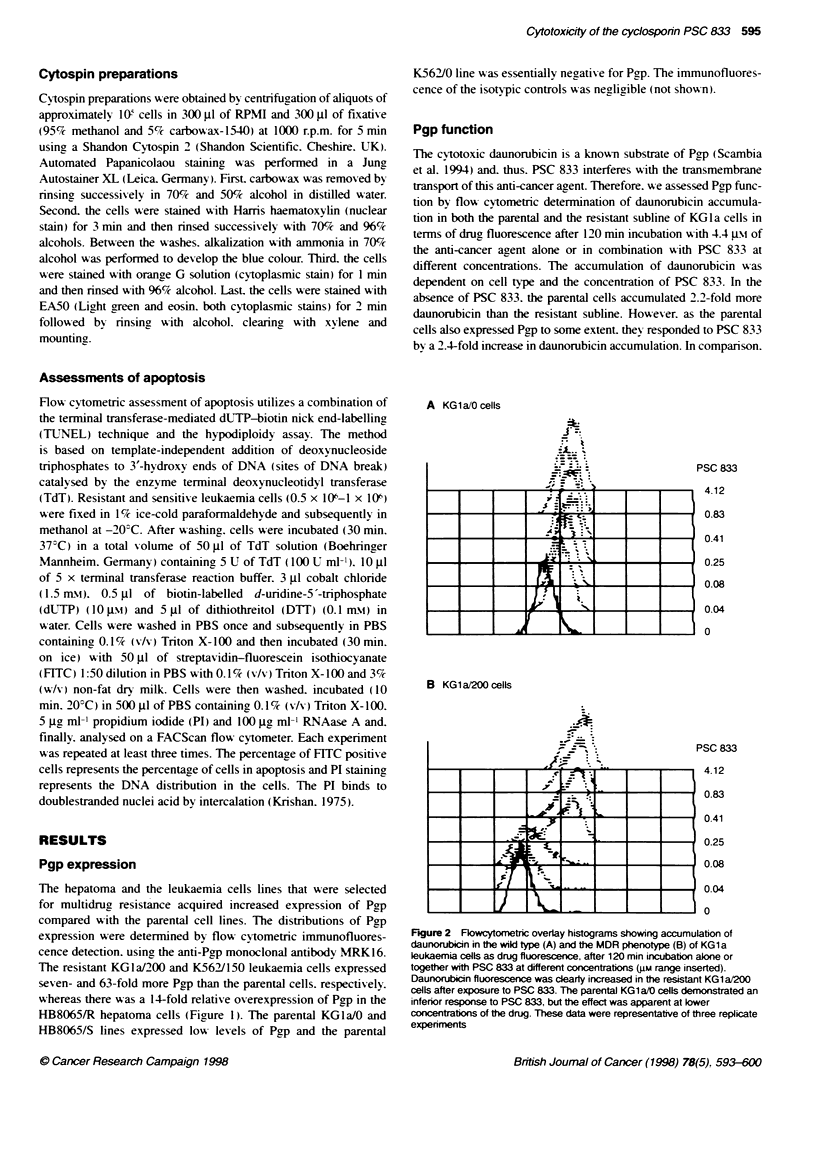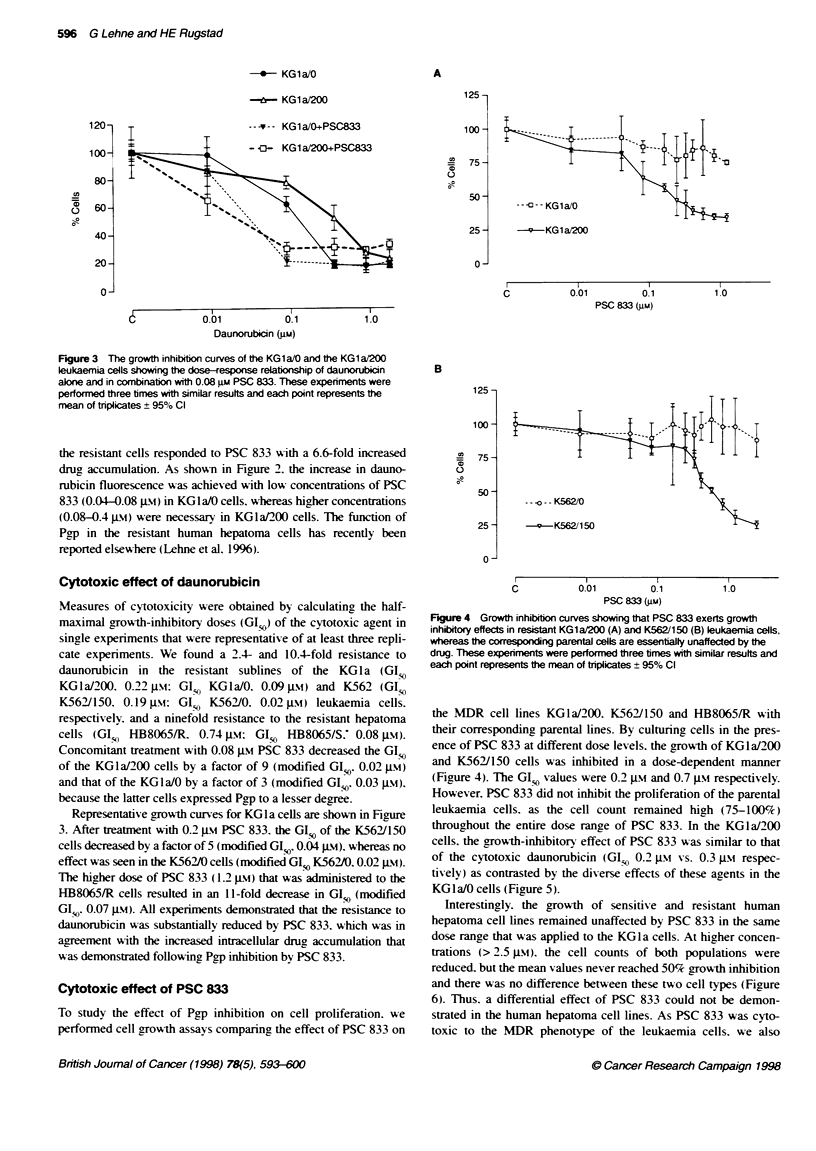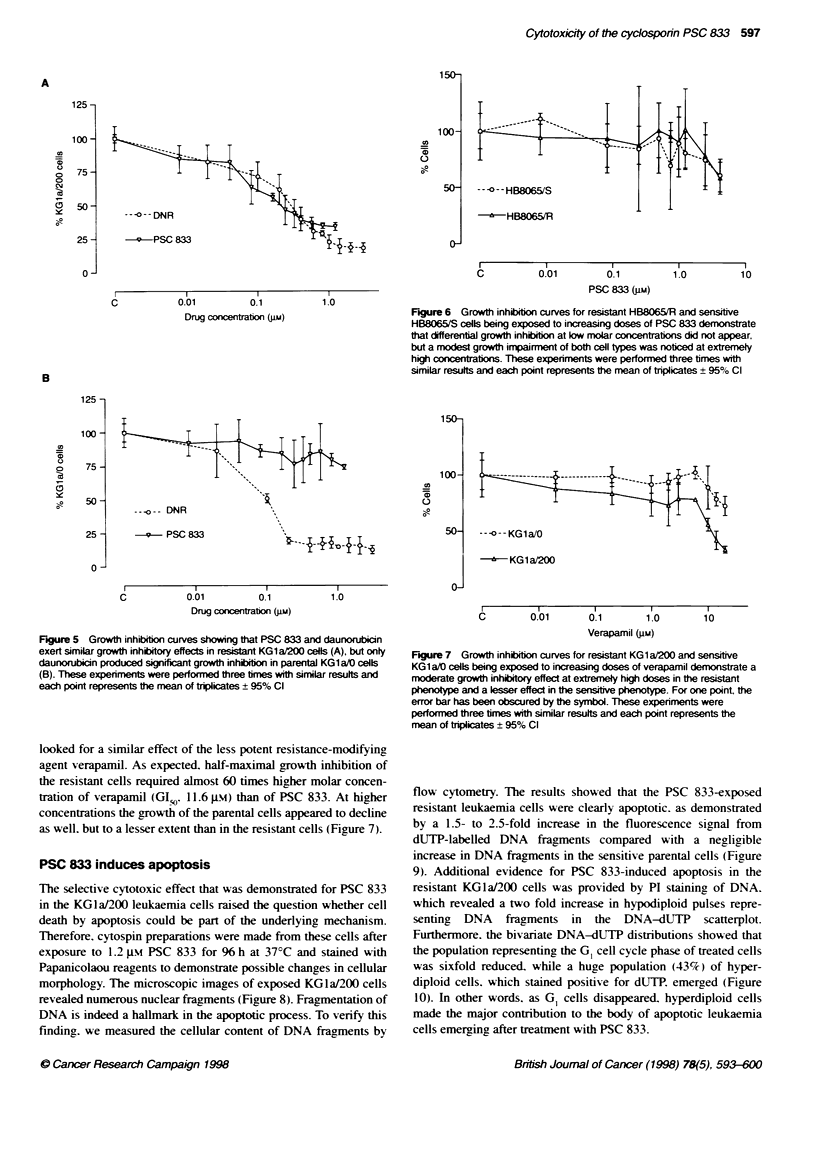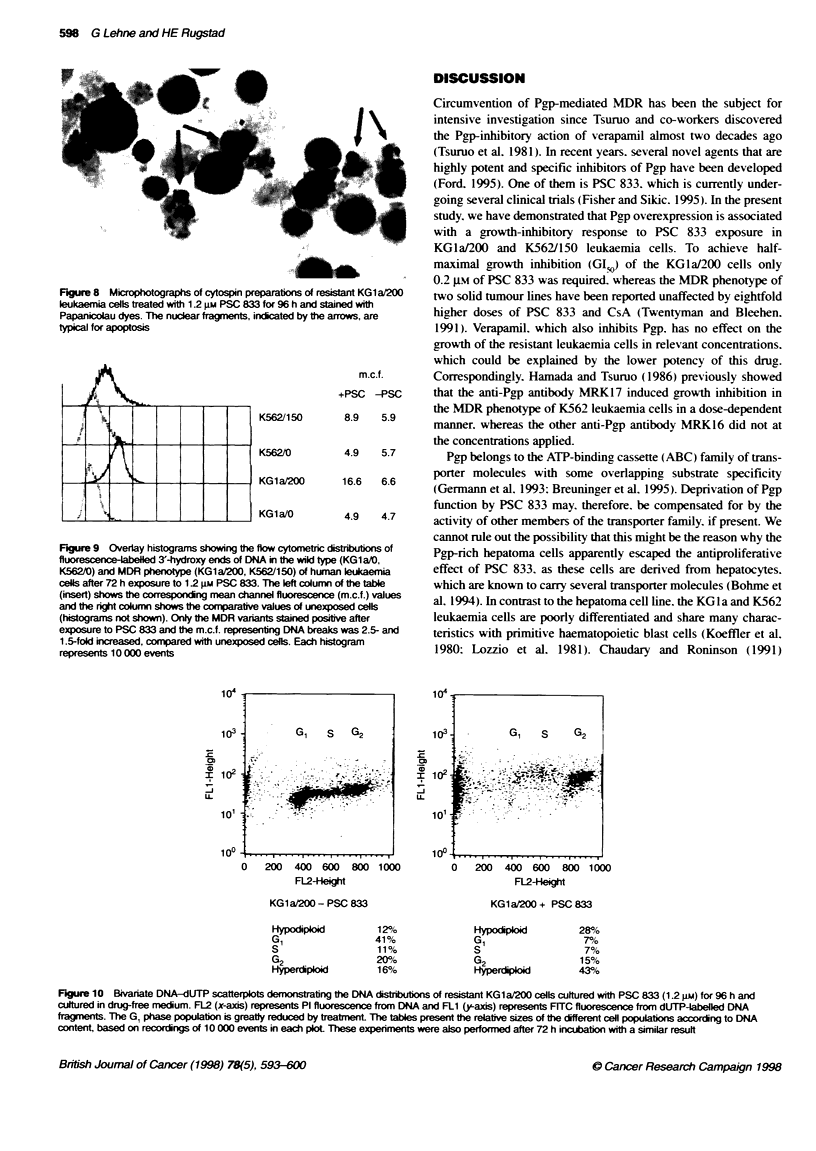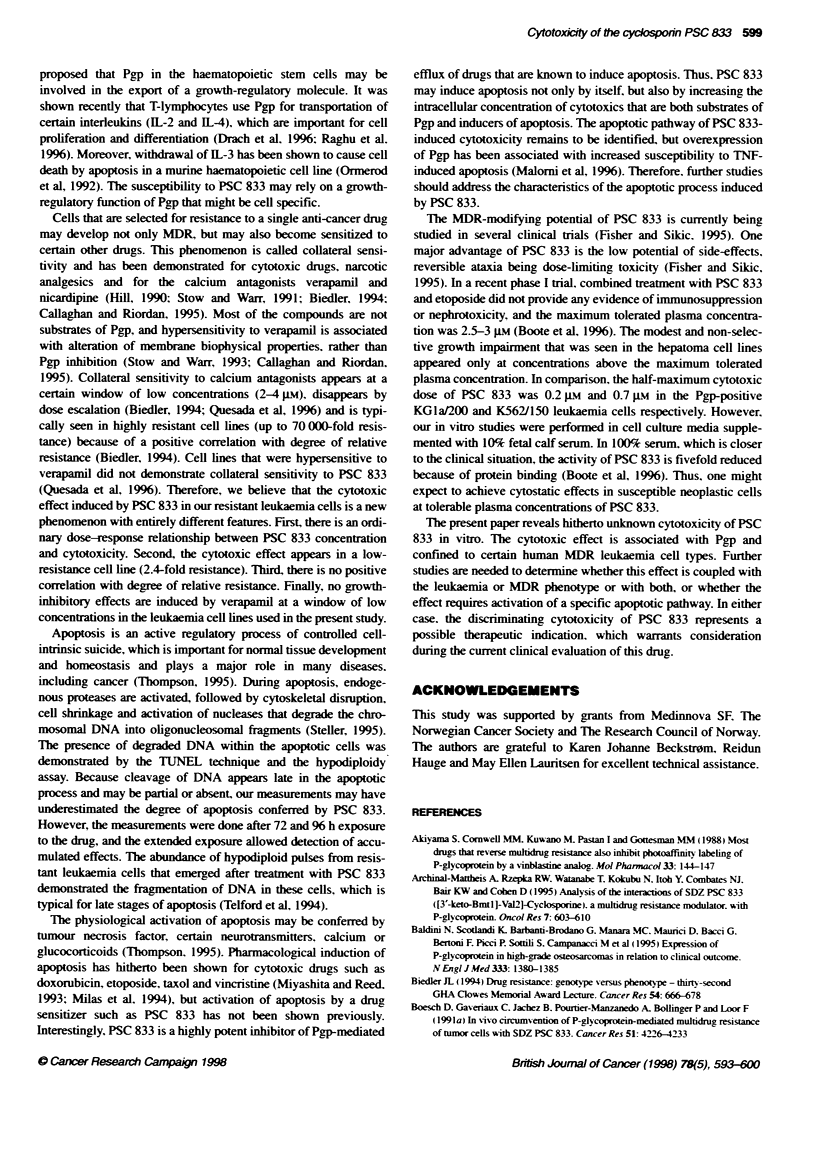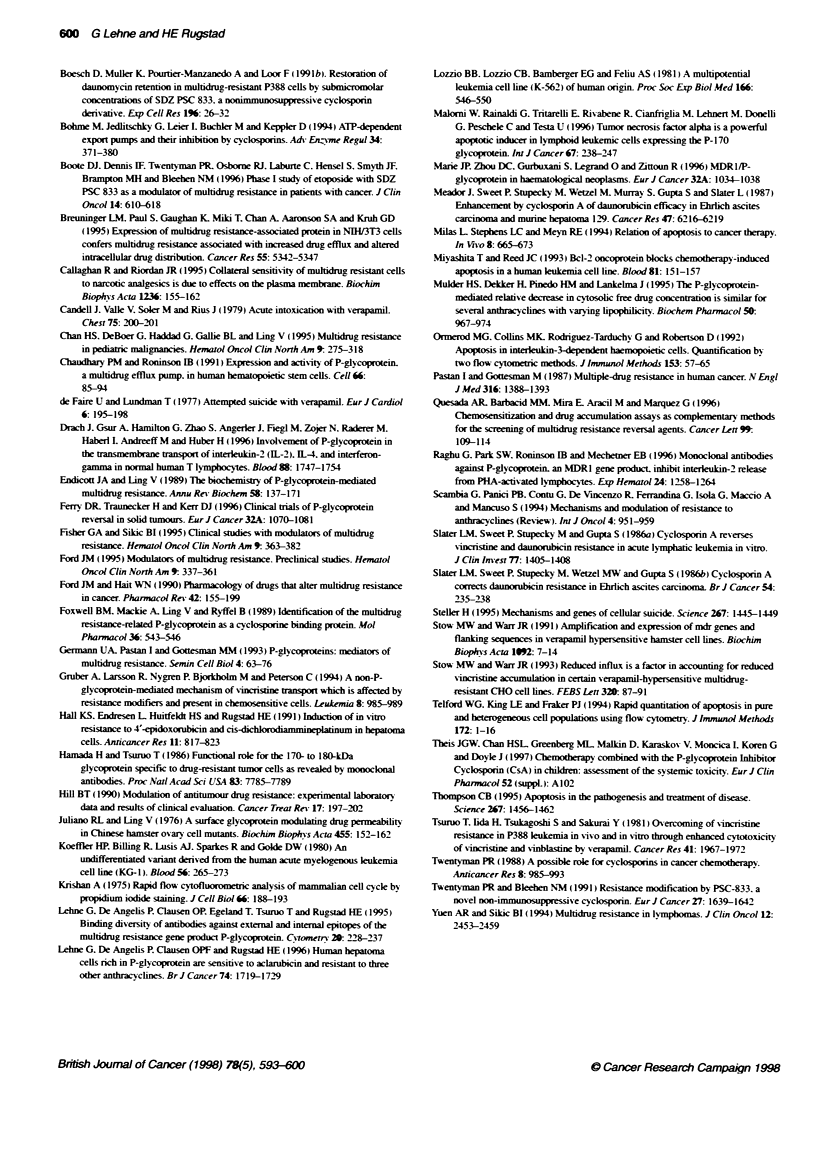# Cytotoxic effect of the cyclosporin PSC 833 in multidrug-resistant leukaemia cells with increased expression of P-glycoprotein.

**DOI:** 10.1038/bjc.1998.546

**Published:** 1998-09

**Authors:** G. Lehne, H. E. Rugstad

**Affiliations:** Department of Clinical Pharmacology, The National Hospital, Rikshospitalet, University of Oslo, Norway.

## Abstract

**Images:**


British Journal of Cancer (1998) 78(5), 593–600
© 1998 Cancer Research Campaign

### Cytotoxic effect of the cyclosporin PSC 833 in multidrug-resistant leukaemia cells with increased expression of P-glycoprotein

G Lehne[1,2], HE Rugstad[1]

[1]Department of Clinical Pharmacology and [2]Institute for Surgical Research, The National Hospital, Rikshospitalet, University of Oslo, Norway

**Summary** Multidrug resistance (MDR) to anti-cancer agents is frequently associated with overexpression of the drug efflux transporter P-glycoprotein (Pgp) in cancer cells, ensuing drug expulsion and maintenance of tolerable intracellular levels of certain cytotoxic drugs. Pgp may also be present in normal tissue, providing protection against toxic substances, but the physiological role of Pgp is not fully understood. Recently, it was shown that Pgp also takes part in the transport of certain growth-regulating cytokines (Drach et al, 1996; Raghu et al, 1996). Therefore, we studied the effect of the highly potent Pgp inhibitor PSC 833 on proliferation of three pairs of MDR and parental human cell lines (HB8065 hepatoma cells, KG1a and K562 leukaemia cells). The MDR phenotypes were characterized by Pgp overexpression, which was demonstrated by flow cytometry using the anti-Pgp antibody MRK16. Electronic cell counting of 72–96 h cultures revealed a dose-dependent antiproliferative effect of PSC 833 in the resistant KG1a/200 and K562/150 cells. The half-maximal growth inhibitory concentrations ($GI_{50}$) were 0.2 μM and 0.7 μM respectively. Exposure to PSC 833 induced cell death by apoptosis in both cell types, as revealed by flow cytometry and detection of 3′-hydroxy ends of DNA (the result of DNA fragmentation associated with apoptosis), by terminal transferase-mediated dUTP-biotin nick end-labelling (TUNEL). Similar effects were not found in the hepatoma cell lines or the parental leukaemia lines. These results demonstrated a discriminating cytotoxicity of PSC 833 in two human leukaemia MDR variants, representing a possible therapeutic indication which warrants consideration during the ongoing clinical evaluation of this drug.

**Keywords:** P-glycoprotein; apoptosis, leukaemia; cyclosporin

Failure to achieve complete and durable responses from cancer chemotherapy is a common clinical problem that limits the curative potential of antineoplastic agents in cancer treatment. Multidrug resistance (MDR) is believed to be a major cause of treatment failure and is frequently associated with overexpression of the multidrug transporter P-glycoprotein (Pgp), which is an integral plasma membrane protein capable of drug expulsion and maintenance of tolerable intracellular levels of certain cytotoxic drugs (Juliano and Ling, 1976; Endicott and Ling, 1989). Pgp has a broad specificity for multiple xenobiotics (Pastan and Gottesman, 1987), many of which are clinically important anti-cancer drugs with diverse structures and mechanisms of action (Mulder et al, 1995). Thus, Pgp expression has been shown to correlate negatively with chemosensitivity and survival in some solid tumours and various haematological malignancies (Yuen and Sikic, 1994; Baldini et al, 1995; Chan et al, 1995; Marie et al, 1996).

Several non-cytotoxic drugs (e.g. calcium channel blockers, calmodulin antagonists, quinolines, cyclosporins) are also putative substrates of Pgp, and some have been shown to inhibit drug efflux competitively and thereby reverse MDR experimentally (Ford and Hait, 1990). The calcium channel blocker verapamil was the first agent that proved to modify MDR in vivo and in vitro (Tsuruo et al, 1981), but, unfortunately, the MDR modifying activity required concentrations that are associated with severe cardiac toxicity in patients (de Faire and Lundman, 1977; Candell et al, 1979). The immunosuppressive agent cyclosporin A (CsA) has been shown to be a highly potent inhibitor of Pgp both in cell lines (Slater et al, 1986*a*; Twentyman, 1988) and in animal models (Slater et al, 1986*b*; Meador et al, 1987). Although CsA inhibits Pgp at clinically relevant concentrations, the immunosuppression induced by this agent may be detrimental to cancer patients. Combined regimens of CsA and chemotherapeutic agents are associated with substantial toxicity and increased hospital admissions for treatment of septicaemia in particular (Theis et al, 1997).

Therefore, the introduction of a novel non-immunosuppressant and highly effective MDR modifying cyclosporin, PSC 833, has provided new perspectives for therapeutic MDR reversal, and the drug is currently undergoing extensive clinical evaluation.

PSC 833 is a cyclosporin D analogue which is tenfold more potent than CsA with respect to MDR reversal (Boesch et al, 1991*a*, 1991*b*). Photolabelling experiments using Pgp-rich membrane fragments have demonstrated that CsA binds to Pgp in vitro (Akiyama et al, 1988; Foxwell et al, 1989). Similarly, PSC 833 has been shown to displace $^3$H-photoaffinity-labelled CsA from Pgp (Archinal-Mattheis et al, 1995) and compete with [$^3$H]vinblastine binding to Pgp at a low molar level, the equilibrium constant, $K_i$, being 35 nM (Ferry et al, 1996). Thus, PSC 833 appears to be suitable for arresting Pgp function in studies of the physiological role of Pgp.

Recently, it was shown that Pgp participates in the transport of interleukins, which are important for proliferation and differentiation of certain cell types (Drach et al, 1996; Raghu et al, 1996). In the present study, we investigated the effect of Pgp inhibition by

*Received 27 June 1997*
*Revised 15 December 1997*
*Accepted 17 February 1998*

*Correspondence to:* G Lehne, Department of Clinical Pharmacology, Rikshospitalet, The National Hospital, N-0027 Oslo, Norway

PSC 833 on the proliferation of the MDR phenotype and the wild type of two pairs of human KG1a and K562 leukaemia cells and a pair of human HB8065 hepatoma cells. The results demonstrate differential effects of PSC 833 on the viability and growth of these separate cell types.

### MATERIALS AND METHODS

### Chemicals

The leukaemia cell lines were propagated in RPMI-1640 medium (Bio Whittaker, Walkersville, MA, USA) and the hepatoma cell lines in Eagle's modified minimum essential medium (EMEM, Bio Whittaker). Both growth media were supplemented with 10% fetal calf serum, L-glutamine (0.05 mM $ml^{-1}$), streptomycin (100 μg $ml^{-1}$), penicillin (100 U $ml^{-1}$), nystatin (40 U $ml^{-1}$) and Hepes (only K562 cell lines). The primary antibody MRK16 (Hamada and Tsuruo, 1986), which was a gift from Professor Takashi Tsuruo (Institute of Molecular and Cellular Biosciences, The University of Tokyo, Japan), reacts with a membrane surface domain of Pgp. The corresponding isotypic control antibody Mouse IgG2a was purchased from Monosan (Uden, The Netherlands). PSC 833 was supplied by Novartis (Basle, Switzerland), verapamil by Knoll (Ludwigshafen, Germany), daunorubicin by Rhône Poulenc Rorer (Vitry, France), vincristine by Eli Lilly (Indianapolis, IN, USA) and epirubicin by Pharmacia (Milan, Italy).

### Cells and culture conditions

Multidrug-resistant sublines of acute myelogenous leukaemia cells, KG1a (American Type Culture Collection, ATCC), human chronic myelogenous leukaemia cells, K562 (ATCC), and human hepatoma cells, HB8065 (ATCC), were selected for our studies. These cell lines were exposed to stepwise increased drug concentrations in the culture medium and, finally, KG1a/200 cells were maintained in medium containing 100 ng $ml^{-1}$ daunorubicin and vincristine (Lehne et al, 1995), K562/150 in medium containing 150 nM vincristine (Gruber et al, 1994) and HB8065/R in medium containing 125 ng $ml^{-1}$ epirubicin (Hall et al, 1991). The parental cell lines (KG1a/0, K562/0 and HB8065/S) and the corresponding resistant sublines were propagated as previously described (Gruber et al, 1994; Lehne et al, 1995).

### Flow cytometry: immunofluorescence assay of Pgp expression

Specific immunoflourescence was obtained by a three-layer staining technique. Cell suspensions were washed with phosphate-buffered saline/bovine serum albumin (PBS/BSA) and incubated on ice for 60 min with MRK16 (25 μg $ml^{-1}$) or Mouse IgG2a (25 μg $ml^{-1}$) in PBS/BSA. The second- and third-layer staining protocols were carried out with 100 μl of biotinylated horse anti-mouse IgG (1:35 dilution in PBS/BSA) and 100 μl of fluorescein isothiocyanate (FITC)-conjugated streptavidin (1:35 dilution in PBS/BSA) for 20–30 min each, respectively, with one PBS/BSA wash between them. Immunofluorescence distributions were generated using a FACScan flow cytometer (Becton Dickinson, San José, CA, USA) with a 15-mW argon ion laser tuned to 488 nm. FITC fluorescence of gated populations was collected through a bandpass filter (FL1, bandwidth 515–545 nm). Data from 10 000 events were collected and calculations of logarithmically amplified fluorescence values were performed in arithmetic mode using the LYSIS (Becton Dickinson) computer program. Each experiment was repeated at least three times.

### Flow cytometry: intracellular accumulation of anthracyclines

The cells were grown in drug-free medium for 24 h prior to flow cytometric analysis. Then the cells were incubated for 120 min at 37°C with daunorubicin (4.4 μM) alone or in combination with PSC 833 (0.04–4.1 μM). Immediately after, the cell samples were placed on ice and daunorubicin fluorescence was acquired by the FACScan flow cytometer using a bandpass filter of 564–606 nm (FL2). Correlated forward angle (a relative measure of cell size) and right angle (a measure of cell granularity) light scatter measurements were generated to exclude dead cells and debris. Analyses were performed as described above. Each experiment was repeated at least three times.

### Cell growth inhibition assay

Approximately $5 \times 10^4$ cells were plated in 16-mm-diameter wells (Costar Corporation, Cambridge, MA, USA) and grown in 1 ml of drug-free medium for the first 24 h. Then the wells were supplemented with the drug or combination of drugs in a certain dose range. At least three replicate cultures were made from each of the dose levels and from untreated controls. The KG1a cell lines were treated for 96 h and the others for 72 h. Harvested cells were counted in a Coulter counter ZM (Coulter Electronics, Luton, UK). The dose level required for 50% inhibition of cell growth ($GI_{50}$) was calculated from linear plots of dose vs cell number. The resistance factor (RF) was defined as the ratio between the $GI_{50}$ values obtained in the resistant and sensitive cells. Modulation of growth inhibition was assessed by co-incubation with PSC 833. The modulating factor (MF) was defined as the ratio between the $GI_{50}$ values of multiresistant cells with and without PSC 833. Each experiment was repeated at least three times.

**Figure 1** Overlay histograms showing the flow cytometric distribution of Pgp immunofluorescence by MRK16 in the wild type and the MDR phenotype of human leukaemia (KG1a, K562) and hepatoma cells (HB8065). The Pgp expression was increased seven-, 14- and 63-fold in the MDR variants of KG1a, HB8065 and K562 cells respectively. The column to the right lists the mean channel fluorescence (m.c.f.) values. These data were representative of three replicate experiments

 © Cancer Research Campaign 1998

### Cytospin preparations

Cytospin preparations were obtained by centrifugation of aliquots of approximately $10^5$ cells in 300 µl of RPMI and 300 µl of fixative (95% methanol and 5% carbowax-1540) at 1000 r.p.m. for 5 min using a Shandon Cytospin 2 (Shandon Scientific, Cheshire, UK). Automated Papanicolaou staining was performed in a Jung Autostainer XL (Leica, Germany). First, carbowax was removed by rinsing successively in 70% and 50% alcohol in distilled water. Second, the cells were stained with Harris haematoxylin (nuclear stain) for 3 min and then rinsed successively with 70% and 96% alcohols. Between the washes, alkalization with ammonia in 70% alcohol was performed to develop the blue colour. Third, the cells were stained with orange G solution (cytoplasmic stain) for 1 min and then rinsed with 96% alcohol. Last, the cells were stained with EA50 (Light green and eosin, both cytoplasmic stains) for 2 min followed by rinsing with alcohol, clearing with xylene and mounting.

### Assessments of apoptosis

Flow cytometric assessment of apoptosis utilizes a combination of the terminal transferase-mediated dUTP–biotin nick end-labelling (TUNEL) technique and the hypodiploidy assay. The method is based on template-independent addition of deoxynucleoside triphosphates to 3′-hydroxy ends of DNA (sites of DNA break) catalysed by the enzyme terminal deoxynucleotidyl transferase (TdT). Resistant and sensitive leukaemia cells ($0.5 \times 10^6$–$1 \times 10^6$) were fixed in 1% ice-cold paraformaldehyde and subsequently in methanol at −20°C. After washing, cells were incubated (30 min, 37°C) in a total volume of 50 µl of TdT solution (Boehringer Mannheim, Germany) containing 5 U of TdT (100 U $ml^{-1}$), 10 µl of 5 × terminal transferase reaction buffer, 3 µl cobalt chloride (1.5 mM), 0.5 µl of biotin-labelled *d*-uridine-5′-triphosphate (dUTP) (10 µM) and 5 µl of dithiothreitol (DTT) (0.1 mM) in water. Cells were washed in PBS once and subsequently in PBS containing 0.1% (v/v) Triton X-100 and then incubated (30 min, on ice) with 50 µl of streptavidin–fluorescein isothiocyanate (FITC) 1:50 dilution in PBS with 0.1% (v/v) Triton X-100 and 3% (w/v) non-fat dry milk. Cells were then washed, incubated (10 min, 20°C) in 500 µl of PBS containing 0.1% (v/v) Triton X-100, 5 µg $ml^{-1}$ propidium iodide (PI) and 100 µg $ml^{-1}$ RNAase A and, finally, analysed on a FACScan flow cytometer. Each experiment was repeated at least three times. The percentage of FITC positive cells represents the percentage of cells in apoptosis and PI staining represents the DNA distribution in the cells. The PI binds to doublestranded nuclei acid by intercalation (Krishan, 1975).

### RESULTS

### Pgp expression

The hepatoma and the leukaemia cells lines that were selected for multidrug resistance acquired increased expression of Pgp compared with the parental cell lines. The distributions of Pgp expression were determined by flow cytometric immunofluorescence detection, using the anti-Pgp monoclonal antibody MRK16. The resistant KG1a/200 and K562/150 leukaemia cells expressed seven- and 63-fold more Pgp than the parental cells, respectively, whereas there was a 14-fold relative overexpression of Pgp in the HB8065/R hepatoma cells (Figure 1). The parental KG1a/0 and HB8065/S lines expressed low levels of Pgp and the parental K562/0 line was essentially negative for Pgp. The immunofluorescence of the isotypic controls was negligible (not shown).

### Pgp function

The cytotoxic daunorubicin is a known substrate of Pgp (Scambia et al, 1994) and, thus, PSC 833 interferes with the transmembrane transport of this anti-cancer agent. Therefore, we assessed Pgp function by flow cytometric determination of daunorubicin accumulation in both the parental and the resistant subline of KG1a cells in terms of drug fluorescence after 120 min incubation with 4.4 µM of the anti-cancer agent alone or in combination with PSC 833 at different concentrations. The accumulation of daunorubicin was dependent on cell type and the concentration of PSC 833. In the absence of PSC 833, the parental cells accumulated 2.2-fold more daunorubicin than the resistant subline. However, as the parental cells also expressed Pgp to some extent, they responded to PSC 833 by a 2.4-fold increase in daunorubicin accumulation. In comparison,

**Figure 2** Flowcytometric overlay histograms showing accumulation of daunorubicin in the wild type (A) and the MDR phenotype (B) of KG1a leukaemia cells as drug fluorescence, after 120 min incubation alone or together with PSC 833 at different concentrations (µM range inserted). Daunorubicin fluorescence was clearly increased in the resistant KG1a/200 cells after exposure to PSC 833. The parental KG1a/0 cells demonstrated an inferior response to PSC 833, but the effect was apparent at lower concentrations of the drug. These data were representative of three replicate experiments

© Cancer Research Campaign 1998

**Figure 3** The growth inhibition curves of the KG1a/0 and the KG1a/200 leukaemia cells showing the dose–response relationship of daunorubicin alone and in combination with 0.08 μM PSC 833. These experiments were performed three times with similar results and each point represents the mean of triplicates ± 95% CI

**Figure 4** Growth inhibition curves showing that PSC 833 exerts growth inhibitory effects in resistant KG1a/200 (A) and K562/150 (B) leukaemia cells, whereas the corresponding parental cells are essentially unaffected by the drug. These experiments were performed three times with similar results and each point represents the mean of triplicates ± 95% CI

the resistant cells responded to PSC 833 with a 6.6-fold increased drug accumulation. As shown in Figure 2, the increase in daunorubicin fluorescence was achieved with low concentrations of PSC 833 (0.04–0.08 μM) in KG1a/0 cells, whereas higher concentrations (0.08–0.4 μM) were necessary in KG1a/200 cells. The function of Pgp in the resistant human hepatoma cells has recently been reported elsewhere (Lehne et al. 1996).

### Cytotoxic effect of daunorubicin

Measures of cytotoxicity were obtained by calculating the half-maximal growth-inhibitory doses ($GI_{50}$) of the cytotoxic agent in single experiments that were representative of at least three replicate experiments. We found a 2.4- and 10.4-fold resistance to daunorubicin in the resistant sublines of the KG1a ($GI_{50}$ KG1a/200, 0.22 μM; $GI_{50}$ KG1a/0, 0.09 μM) and K562 ($GI_{50}$ K562/150, 0.19 μM; $GI_{50}$ K562/0, 0.02 μM) leukaemia cells, respectively, and a ninefold resistance to the resistant hepatoma cells ($GI_{50}$ HB8065/R, 0.74 μM; $GI_{50}$ HB8065/S,' 0.08 μM). Concomitant treatment with 0.08 μM PSC 833 decreased the $GI_{50}$ of the KG1a/200 cells by a factor of 9 (modified $GI_{50}$, 0.02 μM) and that of the KG1a/0 by a factor of 3 (modified $GI_{50}$, 0.03 μM), because the latter cells expressed Pgp to a lesser degree.

Representative growth curves for KG1a cells are shown in Figure 3. After treatment with 0.2 μM PSC 833, the $GI_{50}$ of the K562/150 cells decreased by a factor of 5 (modified $GI_{50}$, 0.04 μM), whereas no effect was seen in the K562/0 cells (modified $GI_{50}$ K562/0, 0.02 μM). The higher dose of PSC 833 (1.2 μM) that was administered to the HB8065/R cells resulted in an 11-fold decrease in $GI_{50}$ (modified $GI_{50}$, 0.07 μM). All experiments demonstrated that the resistance to daunorubicin was substantially reduced by PSC 833, which was in agreement with the increased intracellular drug accumulation that was demonstrated following Pgp inhibition by PSC 833.

### Cytotoxic effect of PSC 833

To study the effect of Pgp inhibition on cell proliferation, we performed cell growth assays comparing the effect of PSC 833 on the MDR cell lines KG1a/200, K562/150 and HB8065/R with their corresponding parental lines. By culturing cells in the presence of PSC 833 at different dose levels, the growth of KG1a/200 and K562/150 cells was inhibited in a dose-dependent manner (Figure 4). The $GI_{50}$ values were 0.2 μM and 0.7 μM respectively. However, PSC 833 did not inhibit the proliferation of the parental leukaemia cells, as the cell count remained high (75–100%) throughout the entire dose range of PSC 833. In the KG1a/200 cells, the growth-inhibitory effect of PSC 833 was similar to that of the cytotoxic daunorubicin ($GI_{50}$ 0.2 μM vs. 0.3 μM respectively) as contrasted by the diverse effects of these agents in the KG1a/0 cells (Figure 5).

Interestingly, the growth of sensitive and resistant human hepatoma cell lines remained unaffected by PSC 833 in the same dose range that was applied to the KG1a cells. At higher concentrations (> 2.5 μM), the cell counts of both populations were reduced, but the mean values never reached 50% growth inhibition and there was no difference between these two cell types (Figure 6). Thus, a differential effect of PSC 833 could not be demonstrated in the human hepatoma cell lines. As PSC 833 was cytotoxic to the MDR phenotype of the leukaemia cells, we also

© Cancer Research Campaign 1998

**Figure 5** Growth inhibition curves showing that PSC 833 and daunorubicin exert similar growth inhibitory effects in resistant KG1a/200 cells (A), but only daunorubicin produced significant growth inhibition in parental KG1a/0 cells (B). These experiments were performed three times with similar results and each point represents the mean of triplicates ± 95% CI

**Figure 6** Growth inhibition curves for resistant HB8065/R and sensitive HB8065/S cells being exposed to increasing doses of PSC 833 demonstrate that differential growth inhibition at low molar concentrations did not appear, but a modest growth impairment of both cell types was noticed at extremely high concentrations. These experiments were performed three times with similar results and each point represents the mean of triplicates ± 95% CI

**Figure 7** Growth inhibition curves for resistant KG1a/200 and sensitive KG1a/0 cells being exposed to increasing doses of verapamil demonstrate a moderate growth inhibitory effect at extremely high doses in the resistant phenotype and a lesser effect in the sensitive phenotype. For one point, the error bar has been obscured by the symbol. These experiments were performed three times with similar results and each point represents the mean of triplicates ± 95% CI

looked for a similar effect of the less potent resistance-modifying agent verapamil. As expected, half-maximal growth inhibition of the resistant cells required almost 60 times higher molar concentration of verapamil ($GI_{50}$, 11.6 μM) than of PSC 833. At higher concentrations the growth of the parental cells appeared to decline as well, but to a lesser extent than in the resistant cells (Figure 7).

### PSC 833 induces apoptosis

The selective cytotoxic effect that was demonstrated for PSC 833 in the KG1a/200 leukaemia cells raised the question whether cell death by apoptosis could be part of the underlying mechanism. Therefore, cytospin preparations were made from these cells after exposure to 1.2 μM PSC 833 for 96 h at 37°C and stained with Papanicolaou reagents to demonstrate possible changes in cellular morphology. The microscopic images of exposed KG1a/200 cells revealed numerous nuclear fragments (Figure 8). Fragmentation of DNA is indeed a hallmark in the apoptotic process. To verify this finding, we measured the cellular content of DNA fragments by flow cytometry. The results showed that the PSC 833-exposed resistant leukaemia cells were clearly apoptotic, as demonstrated by a 1.5- to 2.5-fold increase in the fluorescence signal from dUTP-labelled DNA fragments compared with a negligible increase in DNA fragments in the sensitive parental cells (Figure 9). Additional evidence for PSC 833-induced apoptosis in the resistant KG1a/200 cells was provided by PI staining of DNA, which revealed a two fold increase in hypodiploid pulses representing DNA fragments in the DNA–dUTP scatterplot. Furthermore, the bivariate DNA–dUTP distributions showed that the population representing the $G_1$ cell cycle phase of treated cells was sixfold reduced, while a huge population (43%) of hyperdiploid cells, which stained positive for dUTP, emerged (Figure 10). In other words, as $G_1$ cells disappeared, hyperdiploid cells made the major contribution to the body of apoptotic leukaemia cells emerging after treatment with PSC 833.

**Figure 8** Microphotographs of cytospin preparations of resistant KG1a/200 leukaemia cells treated with 1.2 μM PSC 833 for 96 h and stained with Papanicolau dyes. The nuclear fragments, indicated by the arrows, are typical for apoptosis

| | m.c.f. | |
|---|---|---|
| | +PSC | –PSC |
| K562/150 | 8.9 | 5.9 |
| K562/0 | 4.9 | 5.7 |
| KG1a/200 | 16.6 | 6.6 |
| KG1a/0 | 4.9 | 4.7 |

**Figure 9** Overlay histograms showing the flow cytometric distributions of fluorescence-labelled 3′-hydroxy ends of DNA in the wild type (KG1a/0, K562/0) and MDR phenotype (KG1a/200, K562/150) of human leukaemia cells after 72 h exposure to 1.2 μM PSC 833. The left column of the table (insert) shows the corresponding mean channel fluorescence (m.c.f.) values and the right column shows the comparative values of unexposed cells (histograms not shown). Only the MDR variants stained positive after exposure to PSC 833 and the m.c.f. representing DNA breaks was 2.5- and 1.5-fold increased, compared with unexposed cells. Each histogram represents 10 000 events

### DISCUSSION

Circumvention of Pgp-mediated MDR has been the subject for intensive investigation since Tsuruo and co-workers discovered the Pgp-inhibitory action of verapamil almost two decades ago (Tsuruo et al, 1981). In recent years, several novel agents that are highly potent and specific inhibitors of Pgp have been developed (Ford, 1995). One of them is PSC 833, which is currently undergoing several clinical trials (Fisher and Sikic, 1995). In the present study, we have demonstrated that Pgp overexpression is associated with a growth-inhibitory response to PSC 833 exposure in KG1a/200 and K562/150 leukaemia cells. To achieve half-maximal growth inhibition ($GI_{50}$) of the KG1a/200 cells only 0.2 μM of PSC 833 was required, whereas the MDR phenotype of two solid tumour lines have been reported unaffected by eightfold higher doses of PSC 833 and CsA (Twentyman and Bleehen, 1991). Verapamil, which also inhibits Pgp, has no effect on the growth of the resistant leukaemia cells in relevant concentrations, which could be explained by the lower potency of this drug. Correspondingly, Hamada and Tsuruo (1986) previously showed that the anti-Pgp antibody MRK17 induced growth inhibition in the MDR phenotype of K562 leukaemia cells in a dose-dependent manner, whereas the other anti-Pgp antibody MRK16 did not at the concentrations applied.

Pgp belongs to the ATP-binding cassette (ABC) family of transporter molecules with some overlapping substrate specificity (Germann et al, 1993; Breuninger et al, 1995). Deprivation of Pgp function by PSC 833 may, therefore, be compensated for by the activity of other members of the transporter family, if present. We cannot rule out the possibility that this might be the reason why the Pgp-rich hepatoma cells apparently escaped the antiproliferative effect of PSC 833, as these cells are derived from hepatocytes, which are known to carry several transporter molecules (Bohme et al, 1994). In contrast to the hepatoma cell line, the KG1a and K562 leukaemia cells are poorly differentiated and share many characteristics with primitive haematopoietic blast cells (Koeffler et al, 1980; Lozzio et al, 1981). Chaudary and Roninson (1991)

| KG1a/200 – PSC 833 | |
|---|---|
| Hypodiploid | 12% |
| $G_1$ | 41% |
| S | 11% |
| $G_2$ | 20% |
| Hyperdiploid | 16% |

| KG1a/200 + PSC 833 | |
|---|---|
| Hypodiploid | 28% |
| $G_1$ | 7% |
| S | 7% |
| $G_2$ | 15% |
| Hyperdiploid | 43% |

**Figure 10** Bivariate DNA–dUTP scatterplots demonstrating the DNA distributions of resistant KG1a/200 cells cultured with PSC 833 (1.2 μM) for 96 h and cultured in drug-free medium. FL2 (x-axis) represents PI fluorescence from DNA and FL1 (y-axis) represents FITC fluorescence from dUTP-labelled DNA fragments. The $G_1$ phase population is greatly reduced by treatment. The tables present the relative sizes of the different cell populations according to DNA content, based on recordings of 10 000 events in each plot. These experiments were also performed after 72 h incubation with a similar result

© Cancer Research Campaign 1998

proposed that Pgp in the haematopoietic stem cells may be involved in the export of a growth-regulatory molecule. It was shown recently that T-lymphocytes use Pgp for transportation of certain interleukins (IL-2 and IL-4), which are important for cell proliferation and differentiation (Drach et al, 1996; Raghu et al, 1996). Moreover, withdrawal of IL-3 has been shown to cause cell death by apoptosis in a murine haematopoietic cell line (Ormerod et al, 1992). The susceptibility to PSC 833 may rely on a growth-regulatory function of Pgp that might be cell specific.

Cells that are selected for resistance to a single anti-cancer drug may develop not only MDR, but may also become sensitized to certain other drugs. This phenomenon is called collateral sensitivity and has been demonstrated for cytotoxic drugs, narcotic analgesics and for the calcium antagonists verapamil and nicardipine (Hill, 1990; Stow and Warr, 1991; Biedler, 1994; Callaghan and Riordan, 1995). Most of the compounds are not substrates of Pgp, and hypersensitivity to verapamil is associated with alteration of membrane biophysical properties, rather than Pgp inhibition (Stow and Warr, 1993; Callaghan and Riordan, 1995). Collateral sensitivity to calcium antagonists appears at a certain window of low concentrations (2–4 μM), disappears by dose escalation (Biedler, 1994; Quesada et al, 1996) and is typically seen in highly resistant cell lines (up to 70 000-fold resistance) because of a positive correlation with degree of relative resistance (Biedler, 1994). Cell lines that were hypersensitive to verapamil did not demonstrate collateral sensitivity to PSC 833 (Quesada et al, 1996). Therefore, we believe that the cytotoxic effect induced by PSC 833 in our resistant leukaemia cells is a new phenomenon with entirely different features. First, there is an ordinary dose–response relationship between PSC 833 concentration and cytotoxicity. Second, the cytotoxic effect appears in a low-resistance cell line (2.4-fold resistance). Third, there is no positive correlation with degree of relative resistance. Finally, no growth-inhibitory effects are induced by verapamil at a window of low concentrations in the leukaemia cell lines used in the present study.

Apoptosis is an active regulatory process of controlled cell-intrinsic suicide, which is important for normal tissue development and homeostasis and plays a major role in many diseases, including cancer (Thompson, 1995). During apoptosis, endogenous proteases are activated, followed by cytoskeletal disruption, cell shrinkage and activation of nucleases that degrade the chromosomal DNA into oligonucleosomal fragments (Steller, 1995). The presence of degraded DNA within the apoptotic cells was demonstrated by the TUNEL technique and the hypodiploidy assay. Because cleavage of DNA appears late in the apoptotic process and may be partial or absent, our measurements may have underestimated the degree of apoptosis conferred by PSC 833. However, the measurements were done after 72 and 96 h exposure to the drug, and the extended exposure allowed detection of accumulated effects. The abundance of hypodiploid pulses from resistant leukaemia cells that emerged after treatment with PSC 833 demonstrated the fragmentation of DNA in these cells, which is typical for late stages of apoptosis (Telford et al, 1994).

The physiological activation of apoptosis may be conferred by tumour necrosis factor, certain neurotransmitters, calcium or glucocorticoids (Thompson, 1995). Pharmacological induction of apoptosis has hitherto been shown for cytotoxic drugs such as doxorubicin, etoposide, taxol and vincristine (Miyashita and Reed, 1993; Milas et al, 1994), but activation of apoptosis by a drug sensitizer such as PSC 833 has not been shown previously. Interestingly, PSC 833 is a highly potent inhibitor of Pgp-mediated efflux of drugs that are known to induce apoptosis. Thus, PSC 833 may induce apoptosis not only by itself, but also by increasing the intracellular concentration of cytotoxics that are both substrates of Pgp and inducers of apoptosis. The apoptotic pathway of PSC 833-induced cytotoxicity remains to be identified, but overexpression of Pgp has been associated with increased susceptibility to TNF-induced apoptosis (Malorni et al, 1996). Therefore, further studies should address the characteristics of the apoptotic process induced by PSC 833.

The MDR-modifying potential of PSC 833 is currently being studied in several clinical trials (Fisher and Sikic, 1995). One major advantage of PSC 833 is the low potential of side-effects, reversible ataxia being dose-limiting toxicity (Fisher and Sikic, 1995). In a recent phase I trial, combined treatment with PSC 833 and etoposide did not provide any evidence of immunosuppression or nephrotoxicity, and the maximum tolerated plasma concentration was 2.5–3 μM (Boote et al, 1996). The modest and non-selective growth impairment that was seen in the hepatoma cell lines appeared only at concentrations above the maximum tolerated plasma concentration. In comparison, the half-maximum cytotoxic dose of PSC 833 was 0.2 μM and 0.7 μM in the Pgp-positive KG1a/200 and K562/150 leukaemia cells respectively. However, our in vitro studies were performed in cell culture media supplemented with 10% fetal calf serum. In 100% serum, which is closer to the clinical situation, the activity of PSC 833 is fivefold reduced because of protein binding (Boote et al, 1996). Thus, one might expect to achieve cytostatic effects in susceptible neoplastic cells at tolerable plasma concentrations of PSC 833.

The present paper reveals hitherto unknown cytotoxicity of PSC 833 in vitro. The cytotoxic effect is associated with Pgp and confined to certain human MDR leukaemia cell types. Further studies are needed to determine whether this effect is coupled with the leukaemia or MDR phenotype or with both, or whether the effect requires activation of a specific apoptotic pathway. In either case, the discriminating cytotoxicity of PSC 833 represents a possible therapeutic indication, which warrants consideration during the current clinical evaluation of this drug.

### ACKNOWLEDGEMENTS

This study was supported by grants from Medinnova SF, The Norwegian Cancer Society and The Research Council of Norway. The authors are grateful to Karen Johanne Beckstrøm, Reidun Hauge and May Ellen Lauritsen for excellent technical assistance.

### REFERENCES

Akiyama S, Cornwell MM, Kuwano M, Pastan I and Gottesman MM (1988) Most drugs that reverse multidrug resistance also inhibit photoaffinity labeling of P-glycoprotein by a vinblastine analog. *Mol Pharmacol* 33: 144–147

Archinal-Mattheis A, Rzepka RW, Watanabe T, Kokubu N, Itoh Y, Combates NJ, Bair KW and Cohen D (1995) Analysis of the interactions of SDZ PSC 833 ([3′-keto-Bmt1]-Val2]-Cyclosporine), a multidrug resistance modulator, with P-glycoprotein. *Oncol Res* 7: 603–610

Baldini N, Scotlandi K, Barbanti-Brodano G, Manara MC, Maurici D, Bacci G, Bertoni F, Picci P, Sottili S, Campanacci M et al (1995) Expression of P-glycoprotein in high-grade osteosarcomas in relation to clinical outcome. *N Engl J Med* 333: 1380–1385

Biedler JL (1994) Drug resistance: genotype versus phenotype – thirty-second GHA Clowes Memorial Award Lecture. *Cancer Res* 54: 666–678

Boesch D, Gaveriaux C, Jachez B, Pourtier-Manzanedo A, Bollinger P and Loor F (1991*a*) In vivo circumvention of P-glycoprotein-mediated multidrug resistance of tumor cells with SDZ PSC 833. *Cancer Res* 51: 4226–4233

© Cancer Research Campaign 1998

Boesch D, Muller K, Pourtier-Manzanedo A and Loor F (1991*b*). Restoration of daunomycin retention in multidrug-resistant P388 cells by submicromolar concentrations of SDZ PSC 833, a nonimmunosuppressive cyclosporin derivative. *Exp Cell Res* **196**: 26–32
Bohme M, Jedlitschky G, Leier I, Buchler M and Keppler D (1994) ATP-dependent export pumps and their inhibition by cyclosporins. *Adv Enzyme Regul* **34**: 371–380
Boote DJ, Dennis IF, Twentyman PR, Osborne RJ, Laburte C, Hensel S, Smyth JF, Brampton MH and Bleehen NM (1996) Phase I study of etoposide with SDZ PSC 833 as a modulator of multidrug resistance in patients with cancer. *J Clin Oncol* **14**: 610–618
Breuninger LM, Paul S, Gaughan K, Miki T, Chan A, Aaronson SA and Kruh GD (1995) Expression of multidrug resistance-associated protein in NIH/3T3 cells confers multidrug resistance associated with increased drug efflux and altered intracellular drug distribution. *Cancer Res* **55**: 5342–5347
Callaghan R and Riordan JR (1995) Collateral sensitivity of multidrug resistant cells to narcotic analgesics is due to effects on the plasma membrane. *Biochim Biophys Acta* **1236**: 155–162
Candell J, Valle V, Soler M and Rius J (1979) Acute intoxication with verapamil. *Chest* **75**: 200–201
Chan HS, DeBoer G, Haddad G, Gallie BL and Ling V (1995) Multidrug resistance in pediatric malignancies. *Hematol Oncol Clin North Am* **9**: 275–318
Chaudhary PM and Roninson IB (1991) Expression and activity of P-glycoprotein, a multidrug efflux pump, in human hematopoietic stem cells. *Cell* **66**: 85–94
de Faire U and Lundman T (1977) Attempted suicide with verapamil. *Eur J Cardiol* **6**: 195–198
Drach J, Gsur A, Hamilton G, Zhao S, Angerler J, Fiegl M, Zojer N, Raderer M, Haberl I, Andreeff M and Huber H (1996) Involvement of P-glycoprotein in the transmembrane transport of interleukin-2 (IL-2), IL-4, and interferon-gamma in normal human T lymphocytes. *Blood* **88**: 1747–1754
Endicott JA and Ling V (1989) The biochemistry of P-glycoprotein-mediated multidrug resistance. *Annu Rev Biochem* **58**: 137–171
Ferry DR, Traunecker H and Kerr DJ (1996) Clinical trials of P-glycoprotein reversal in solid tumours. *Eur J Cancer* **32A**: 1070–1081
Fisher GA and Sikic BI (1995) Clinical studies with modulators of multidrug resistance. *Hematol Oncol Clin North Am* **9**: 363–382
Ford JM (1995) Modulators of multidrug resistance. Preclinical studies. *Hematol Oncol Clin North Am* **9**: 337–361
Ford JM and Hait WN (1990) Pharmacology of drugs that alter multidrug resistance in cancer. *Pharmacol Rev* **42**: 155–199
Foxwell BM, Mackie A, Ling V and Ryffel B (1989) Identification of the multidrug resistance-related P-glycoprotein as a cyclosporine binding protein. *Mol Pharmacol* **36**: 543–546
Germann UA, Pastan I and Gottesman MM (1993) P-glycoproteins: mediators of multidrug resistance. *Semin Cell Biol* **4**: 63–76
Gruber A, Larsson R, Nygren P, Bjorkholm M and Peterson C (1994) A non-P-glycoprotein-mediated mechanism of vincristine transport which is affected by resistance modifiers and present in chemosensitive cells. *Leukemia* **8**: 985–989
Hall KS, Endresen L, Huitfeldt HS and Rugstad HE (1991) Induction of in vitro resistance to 4′-epidoxorubicin and cis-dichlorodiammineplatinum in hepatoma cells. *Anticancer Res* **11**: 817–823
Hamada H and Tsuruo T (1986) Functional role for the 170- to 180-kDa glycoprotein specific to drug-resistant tumor cells as revealed by monoclonal antibodies. *Proc Natl Acad Sci USA* **83**: 7785–7789
Hill BT (1990) Modulation of antitumour drug resistance: experimental laboratory data and results of clinical evaluation. *Cancer Treat Rev* **17**: 197–202
Juliano RL and Ling V (1976) A surface glycoprotein modulating drug permeability in Chinese hamster ovary cell mutants. *Biochim Biophys Acta* **455**: 152–162
Koeffler HP, Billing R, Lusis AJ, Sparkes R and Golde DW (1980) An undifferentiated variant derived from the human acute myelogenous leukemia cell line (KG-1). *Blood* **56**: 265–273
Krishan A (1975) Rapid flow cytofluorometric analysis of mammalian cell cycle by propidium iodide staining. *J Cell Biol* **66**: 188–193
Lehne G, De Angelis P, Clausen OP, Egeland T, Tsuruo T and Rugstad HE (1995) Binding diversity of antibodies against external and internal epitopes of the multidrug resistance gene product P-glycoprotein. *Cytometry* **20**: 228–237
Lehne G, De Angelis P, Clausen OPF and Rugstad HE (1996) Human hepatoma cells rich in P-glycoprotein are sensitive to aclarubicin and resistant to three other anthracyclines. *Br J Cancer* **74**: 1719–1729
Lozzio BB, Lozzio CB, Bamberger EG and Feliu AS (1981) A multipotential leukemia cell line (K-562) of human origin. *Proc Soc Exp Biol Med* **166**: 546–550
Malorni W, Rainaldi G, Tritarelli E, Rivabene R, Cianfriglia M, Lehnert M, Donelli G, Peschele C and Testa U (1996) Tumor necrosis factor alpha is a powerful apoptotic inducer in lymphoid leukemic cells expressing the P-170 glycoprotein. *Int J Cancer* **67**: 238–247
Marie JP, Zhou DC, Gurbuxani S, Legrand O and Zittoun R (1996) MDR1/P-glycoprotein in haematological neoplasms. *Eur J Cancer* **32A**: 1034–1038
Meador J, Sweet P, Stupecky M, Wetzel M, Murray S, Gupta S and Slater L (1987) Enhancement by cyclosporin A of daunorubicin efficacy in Ehrlich ascites carcinoma and murine hepatoma 129. *Cancer Res* **47**: 6216–6219
Milas L, Stephens LC and Meyn RE (1994) Relation of apoptosis to cancer therapy. *In Vivo* **8**: 665–673
Miyashita T and Reed JC (1993) Bcl-2 oncoprotein blocks chemotherapy-induced apoptosis in a human leukemia cell line. *Blood* **81**: 151–157
Mulder HS, Dekker H, Pinedo HM and Lankelma J (1995) The P-glycoprotein-mediated relative decrease in cytosolic free drug concentration is similar for several anthracyclines with varying lipophilicity. *Biochem Pharmacol* **50**: 967–974
Ormerod MG, Collins MK, Rodriguez-Tarduchy G and Robertson D (1992) Apoptosis in interleukin-3-dependent haemopoietic cells. Quantification by two flow cytometric methods. *J Immunol Methods* **153**: 57–65
Pastan I and Gottesman M (1987) Multiple-drug resistance in human cancer. *N Engl J Med* **316**: 1388–1393
Quesada AR, Barbacid MM, Mira E, Aracil M and Marquez G (1996) Chemosensitization and drug accumulation assays as complementary methods for the screening of multidrug resistance reversal agents. *Cancer Lett* **99**: 109–114
Raghu G, Park SW, Roninson IB and Mechetner EB (1996) Monoclonal antibodies against P-glycoprotein, an MDR1 gene product, inhibit interleukin-2 release from PHA-activated lymphocytes. *Exp Hematol* **24**: 1258–1264
Scambia G, Panici PB, Contu G, De Vincenzo R, Ferrandina G, Isola G, Maccio A and Mancuso S (1994) Mechanisms and modulation of resistance to anthracyclines (Review). *Int J Oncol* **4**: 951–959
Slater LM, Sweet P, Stupecky M and Gupta S (1986*a*) Cyclosporin A reverses vincristine and daunorubicin resistance in acute lymphatic leukemia in vitro. *J Clin Invest* **77**: 1405–1408
Slater LM, Sweet P, Stupecky M, Wetzel MW and Gupta S (1986*b*) Cyclosporin A corrects daunorubicin resistance in Ehrlich ascites carcinoma. *Br J Cancer* **54**: 235–238
Steller H (1995) Mechanisms and genes of cellular suicide. *Science* **267**: 1445–1449
Stow MW and Warr JR (1991) Amplification and expression of mdr genes and flanking sequences in verapamil hypersensitive hamster cell lines. *Biochim Biophys Acta* **1092**: 7–14
Stow MW and Warr JR (1993) Reduced influx is a factor in accounting for reduced vincristine accumulation in certain verapamil-hypersensitive multidrug-resistant CHO cell lines. *FEBS Lett* **320**: 87–91
Telford WG, King LE and Fraker PJ (1994) Rapid quantitation of apoptosis in pure and heterogeneous cell populations using flow cytometry. *J Immunol Methods* **172**: 1–16
Theis JGW, Chan HSL, Greenberg ML, Malkin D, Karaskov V, Moncica I, Koren G and Doyle J (1997) Chemotherapy combined with the P-glycoprotein Inhibitor Cyclosporin (CsA) in children: assessment of the systemic toxicity. *Eur J Clin Pharmacol* **52** (suppl.): A102
Thompson CB (1995) Apoptosis in the pathogenesis and treatment of disease. *Science* **267**: 1456–1462
Tsuruo T, Iida H, Tsukagoshi S and Sakurai Y (1981) Overcoming of vincristine resistance in P388 leukemia in vivo and in vitro through enhanced cytotoxicity of vincristine and vinblastine by verapamil. *Cancer Res* **41**: 1967–1972
Twentyman PR (1988) A possible role for cyclosporins in cancer chemotherapy. *Anticancer Res* **8**: 985–993
Twentyman PR and Bleehen NM (1991) Resistance modification by PSC-833, a novel non-immunosuppressive cyclosporin. *Eur J Cancer* **27**: 1639–1642
Yuen AR and Sikic BI (1994) Multidrug resistance in lymphomas. *J Clin Oncol* **12**: 2453–2459

© Cancer Research Campaign 1998